# Development and Evaluation of Antimicrobial and Modulatory Activity of Inclusion Complex of *Euterpe oleracea* Mart Oil and β-Cyclodextrin or HP-β-Cyclodextrin

**DOI:** 10.3390/ijms21030942

**Published:** 2020-01-31

**Authors:** Thalita Sévia Soares de Almeida Magalhães, Pollyana Cristina de Oliveira Macedo, Stephany Yumi Kawashima Pacheco, Sofia Santos da Silva, Euzébio Guimarães Barbosa, Rayanne Rocha Pereira, Roseane Maria Ribeiro Costa, José Otávio Carréra Silva Junior, Marília Andreza da Silva Ferreira, José Cezário de Almeida, Pedro José Rolim Neto, Attilio Converti, Ádley Antonini Neves de Lima

**Affiliations:** 1 Department of Pharmacy, Laboratório Escola de Farmácia Industrial, Federal University of Rio Grande do Norte, Natal, RN 59012-570, Brazil; thalitasevia22@gmail.com (T.S.S.d.A.M.); macedopollyanax@gmail.com (P.C.d.O.M.); teh.pacheco@hotmail.com (S.Y.K.P.); 2 Department of Pharmacy, Laboratório de Química Farmacêutica Computacional, Federal University of Rio Grande do Norte, Natal, RN 59012-570, Brazil; sofiassilvam@hotmail.com (S.S.d.S.); euzebiogb@gmail.com (E.G.B.); 3 Department of Pharmacy, Laboratório de Pesquisa e Desenvolvimento Farmacêutico e Cosmético, Federal University of Pará, Pará, PA 66075110, Brazil; rayannerocha548@gmail.com (R.R.P.); roseribeiro01@yahoo.com.br (R.M.R.C.); carrera@ufpa.br (J.O.C.S.J.); 4 Department of Nursing, Laboratorio de Microbiologia, Parasitologia and Patologia, Federal University of Campina Grande, Paraíba, PB 58900000, Brazil; marilia.andreza.masf@gmail.com (M.A.d.S.F.); cezarioja@gmail.com (J.C.d.A.); 5 Department of Pharmacy, Laboratory of Medical Technology, Federal University of Pernambuco, Recife, PE 50740-521, Brazil; rolim.pedro@gmail.com; 6 Dipartimento of Civil, Chemical and Environmental Engineering, Pole of Chemical Engineering, Genoa University, I-16145 Genoa, Italy; converti@unige.it

**Keywords:** *Euterpe oleracea* Mart, cyclodextrins, inclusion complexes, antibacterial activity, modulatory activity

## Abstract

The development of inclusion complexes is used to encapsulate nonpolar compounds and improve their physicochemical characteristics. This study aims to develop complexes made up of *Euterpe oleracea* Mart oil (EOO) and β-cyclodextrin (β-CD) or hydroxypropyl-β-cyclodextrin (HP-β-CD) by either kneading (KND) or slurry (SL). Complexes were analyzed by molecular modeling, Fourier-transform infrared spectroscopy, scanning electron microscopy, powder X-ray diffraction, thermogravimetry analysis and differential scanning calorimetry. The antibacterial activity was expressed as Minimum Inhibitory Concentration (MIC), and the antibiotic resistance modulatory activity as subinhibitory concentration (MIC/8) against *Escherichia coli*, *Streptomyces aureus*, *Pseudomonas aeruginosa* and *Enterococcus faecalis*. Inclusion complexes with β-CD and HP-β-CD were confirmed, and efficiency was proven by an interaction energy between oleic acid and β-CD of −41.28 ± 0.57 kJ/mol. MIC values revealed higher antibacterial activity of complexes compared to the isolated oil. The modulatory response of EOO and EOO-β-CD prepared by KND as well as of EOO-β-CD and EOO-HP-β-CD prepared by SL showed a synergistic effect with ampicillin against *E. coli*, whereas it was not significant with the other drugs tested, maintaining the biological response of antibiotics. The antimicrobial response exhibited by the complexes is of great significance because it subsidizes studies for the development of new pharmaceutical forms.

## 1. Introduction

Vegetable oils are a target of studies due to their many pharmacological applications. According to Martins da Silva et al. [1], the chemical constituents present in a variety of oils exhibit different biological activities. The resin oil of *Copaifera multijuga*, commonly used as an anti-inflammatory [2], the essential oil of *Ocimum basilicum*, applied as an antihyperalgesic [3], and the *Allium sativum* oil, displaying antioxidant and antimicrobial activities [4] similar to those of *Euterpe oleracea* oil [5,6], are a few examples.

*E. oleracea* Mart oil (EOO), popularly known as açai oil, is largely produced in Brazil. Rich in long-chain unsaturated fatty acids, its major constituent is oleic acid [7,8], which according to Souza [9] represents 54.32% of the lipidic fraction, followed by palmitic acid (30%), linoleic acid (5.9%) and lower concentrations of palmitoleic, vaccenic and stearic acids. Among the phytosteroids present in the oil, the most important are β-sitosperol, stigmasterol and campesterol. Their ability to improve cell metabolism and reduce inflammatory processes are the reasons why these compounds are widely used by the cosmetic industry to prevent skin aging [10].

Recently, Marques et al. [11], who phytochemically characterized EOO, reported vanillic, palmitic, γ-linolenic, linoleic, oleic, cinnamic, caffeic, protocatechuic, ferulic and syringic acids and the flavonoids quercetin and kaempferol rutinoside as the main phytochemical components of the oil. This variety of constituents may justify the biological activities of açai oil already described [12,13,14,15]. Different studies have shown that EOO has antidiarrheal [16], anti-inflammatory and antinociceptive [8] actions, an inhibitory effect against *Staphylococcus aureus* [6], antiproliferative action [17], and an antiatherogenic effect [9]. The absence of cytotoxicity and genotoxicity was also described [11].

Despite the wide range of promising pharmacological properties, oils are poorly used by the pharmaceutical industry due to their low solubility. To solve this problem, drugs may be complexed with different compounds that are often able to improve their physicochemical properties. Among these, cyclodextrins (CD) are the most frequently used [3,18] followed by polymers such as polyethylene glycol, polyethylene oxide, polyvinyl alcohol and polyvinylpyrrolidone [19].

CD have been used as a pharmaceutical excipient able to increase the solubility of drugs. These cyclic oligosaccharides also exhibit stable physicochemical characteristics capable of protecting host molecules from degradation in the gastrointestinal tract and against oxidation, provide photo and thermal stability, and reduce physiological effects, undesirable organoleptic characteristics, drug volatility and toxicity [20,21,22,23,24,25].

In order to obtain oils with suitable physicochemical characteristics and to improve their functions and pharmacological applications, formulations containing inclusion complexes are being developed, where the product is chemically stable because its constituents are protected by reducing the exposure to environmental and microbiological factors. In addition to these benefits, the formation of inclusion complexes with EOO may improve their solubility, promote oxidative stability against photosensitivity or temperature effects, and especially prevent hydrolytic rancidity.

This work aims to develop inclusion complexes of *E. oleracea* Mart oil with β-cyclodextrin and hydroxypropyl-β-cyclodextrin prepared by kneading and slurry, in order to improve oil physicochemical properties, solubility, stability and antimicrobial activity. The formation of inclusion complexes and the interaction between oleic acid (the main oil component) and cyclodextrins was investigated by Fourier-transform infrared spectroscopy, scanning electron microscopy, powder X-ray diffraction, thermogravimetric analysis, and differential scanning calorimetry. The antibacterial and modulatory activity of the inclusion complexes was tested against standard target bacterial strains using the microdilution test.

## 2. Results and Discussion

### 2.1. Chemical Characterization of Euterpe oleracea Mart Oil

The fatty acid profile determined using gas chromatography (Table 1) shows that the major EOO constituents are oleic (47.58%), palmitic (24.06%) and linoleic (13.58%) acids, confirming the results reported by Do Nascimento et al. [26], while palmitoleic (6.94%), myristic 14:0 (1.75%) and lauric (4.77%) acids were detected in lower contents. This means that approximately 70% of fatty acids are unsaturated, namely oleic acid (18:1), still classified as omega-9, linoleic acid (18:2), characterized as an essential fatty acid capable of being metabolized into omega-6, and palmitoleic acid (16:1). The presence of these compounds is consistent with the antiatherogenic power of this oil [9].

### 2.2. Determination of the Interaction Energy

**Figure 1:**
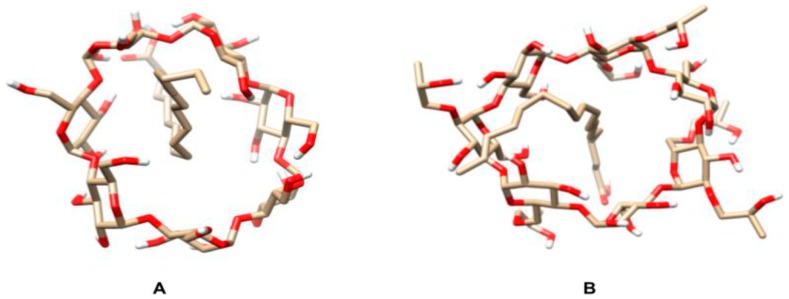
Schematic representation of the inclusion complexes of oleic acid with β-cyclodextrin (β-CD) and hydroxy-propyl-β-cyclodextrin (HP-β-CD). (**A**) β-CD-oleic acid and (**B**) HP-β-CD-oleic acid, with interaction energies of −41.28 ± 0.57 kJ/mol and 25.15 ± 0.44 kJ/mol, respectively. Images were obtained using the UCSF-Chimera software [31].

Apolar ligands of appropriate size tend to replace water molecules bound to cyclodextrin hydroxyl groups in solution, forming more stable and less stressed systems called inclusion complexes [27,28,29,30]. To investigate the possible formation of such complexes between oleic acid, i.e., the major açai oil component, and two different cyclodextrins, a molecular dynamic study was carried out by the Linear Interaction Energy methodology. Figure 1 shows the positions at which the binder was positioned on both cyclodextrins. The position of the binder within both cyclodextrins slightly interfered with the scores obtained after the simulations, with the interaction energies (Δ*G*) of the β-CD:oleic acid and HP-β-CD:oleic acid systems being −41.28 ± 0.57 and 25.15 ± 0.44 kJ/mol, respectively.

The formation of inclusion complexes is directly influenced by Van der Waals forces due to the lipophilic nature of cyclodextrin cavity [32]. Since β-CD chemical affinity for oleic acid is greater than that of HP-β-CD, β-CD:oleic acid inclusion complexes are expected to form more easily and be more stable than the HP-β-CD:oleic acid ones.

Not all molecules in a compound form inclusion complexes, and EOO consists primarily of fatty acids. According to Leclercq [33], β-CD are excellently suited to solubilize phospholipid and easily include them in cyclodextrins, as they are also composed of fatty acids. This complexation, which is characteristic of cyclodextrins, makes encapsulated compounds stable and improves the physicochemical properties of the substance.

The formation of inclusion complexes is influenced by CD-linked substituents and, our findings are in accordance with and Arima [34]. Hydrophilic substituents, such as 2-hydroxypropyl, 3-hydroxypropyl, and maltosil, for example, reduce hemolytic activity compared to original CDs, while lipophilic substituents (e.g., methylated CDs) demonstrate stronger hemolytic activities [34]. This hemolysis is attributed to the complexation capacity of the CD and fatty acids and the removal of this constituent from the erythrocyte membrane.

### 2.3. Physicochemical Characterization of Inclusion Complexes

#### 2.3.1. Fourier Transform Infrared Spectroscopy (FT-IR)

**Figure 2:**
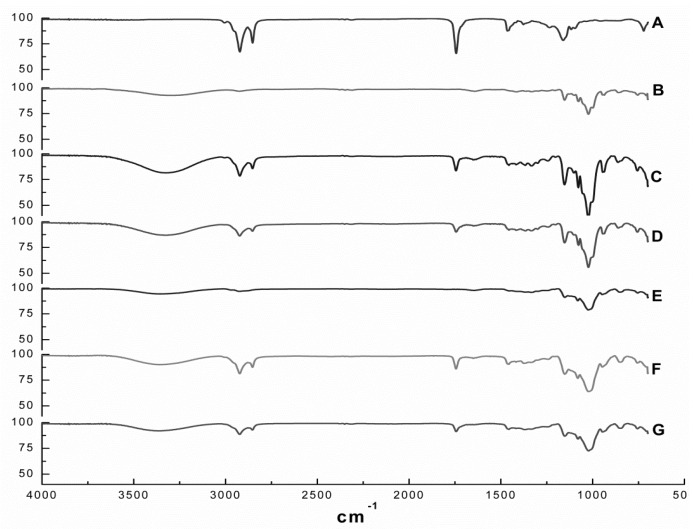
FT-IR spectra of *E. oleracea* oil (EOO) (**A**), β-cyclodextrin (**B**), EOO-β-cyclodextrin inclusion complexes prepared by kneading (**C**) and slurry (**D**), hydroxy-propyl-β-cyclodextrin (**E**), and EOO-hydroxy-propyl-β-cyclodextrin inclusion complexes prepared by kneading (**F**) and slurry (**G**).

Figure 2 depicts the FT-IR spectra of EOO, β-CD, HP-β-CD and the EOO-β-CD and EOO-HP-β-CD inclusion complexes obtained by either kneading (KND) or slurry (SL). The EOO spectrum (Figure 2A) showed relevant bands at wavenumbers of 2924-2849, 1745, 1458 and 1161 cm^−1^ that can be assigned to C–H stretching vibrations, C=O stretching vibrations, C=C asymmetric deformation and C–O stretching vibrations, respectively, while the β-CD spectrum (Figure 2B) showed others at 3302, 1155 and 1024 cm^−1^, corresponding to O–H stretching vibrations, C–O asymmetric deformation and C–O–C symmetric stretching vibrations, thereby confirming the results of Pinheiro et al. [2]. As expected, the HP-β-CD spectrum (Figure 2E) exhibited the same characteristic bands detected for β-CD, besides the additional band at 2918 cm^−1^ corresponding to C–H stretching vibrations, confirming the observations by Medarević et al. [35].

The spectrum of the EOO-β-CD inclusion complex prepared by KND (Figure 2C) showed the typical O–H vibration band at 3319 cm^−1^ and an intensified C–O–C symmetric stretching vibrations band at 1024 cm^−1^, which are characteristic of β-CD molecule, as well as reduced intensity or even suppression of those characteristic of EOO. A similar behavior was observed for the same inclusion complex prepared by SL (Figure 2D), with a reduction in the intensity of bands at 3330, 1155, 1024, 2924, 2855, 1745 and 1453 cm^−1^. EOO-HP-β-CD complexes prepared by both techniques exhibited qualitatively identical spectra, with the characteristic HP-β-CD bands at 3358 and 1018 cm^−1^ corresponding to O–H and C–O–C vibrations, respectively, while the EOO characteristic bands had decreased intensity (Figure 2F,G).

Since this analytical technique detects the vibrational frequency of chemical bonds, the decreased intensity and/or absence of EOO characteristic bands in the spectra of all preparations indicates an interaction between oil and cyclodextrins that constitutes an indirect proof of inclusion complexes formation [36]. Although no significant difference could be detected between spectra of inclusion complexes prepared by kneading or slurry, the latter exhibited patterns more similar to those of their respective cyclodextrins and less pronounced oil characteristic bands.

#### 2.3.2. Scanning Electron Microscopy (SEM)

**Figure 3:**
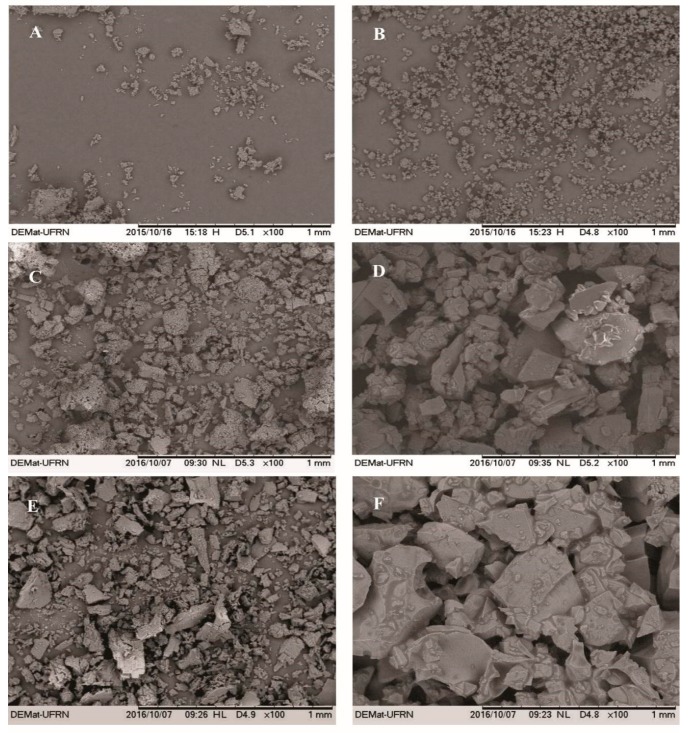
SEM micrographs of β-cyclodextrin (**A**), hydroxy-propyl-β-cyclodextrin (**B**), EOO-β-cyclodextrin inclusion complexes prepared by kneading (**C**) and slurry (**E**), and EOO-hydroxy-propyl-β-cyclodextrin inclusion complexes prepared by kneading (**D**) and slurry (**F**). Images were taken at a magnification of 100×.

Figure 3 shows the micrographs of β-CD, HP-β-CD, EOO-β-CD and EOO-HP-β-CD. Both EOO-β-CD complexes prepared by kneading (Figure 3C) and slurry (Figure 3E) displayed an amorphous state, especially the former, with only very small differences between them. On the other hand, particle appearance, shape and size were quite different between EOO-HP-β-CD complexes, in that, those obtained by SL displayed an even larger difference than those obtained by KND compared to HP-β-CD. As suggested by Bulani et al. [37], such modifications in particle shape and aspect may be taken as a proof of the formation of inclusion complex with CD, thereby confirming the insights emerged from FT-IR spectroscopy.

#### 2.3.3. Powder X-ray Diffraction (PXRD)

**Figure 4:**
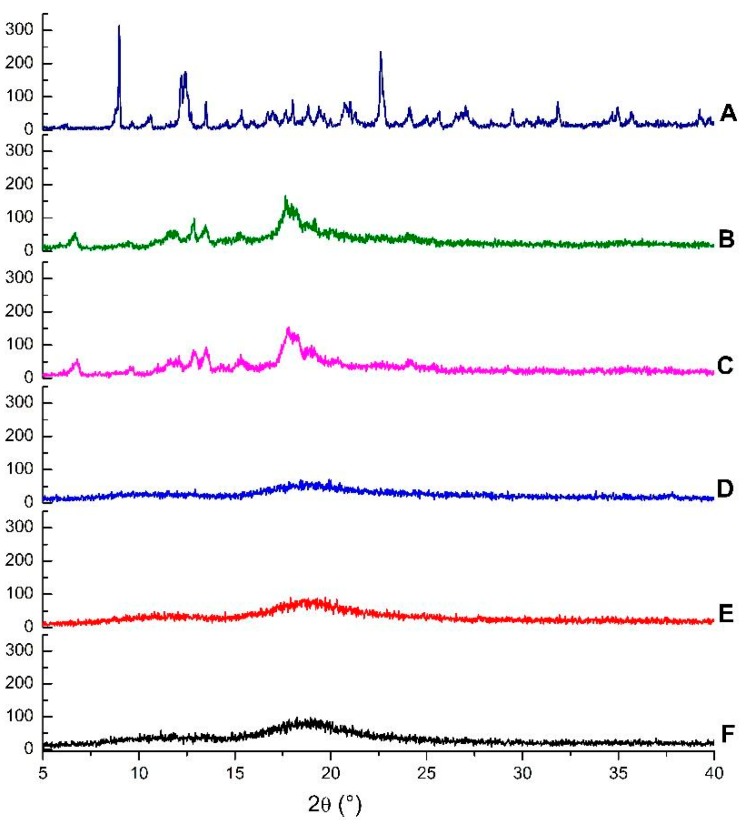
Diffractograms of β-cyclodextrin (**A**), EOO-β-cyclodextrin inclusion complexes prepared by kneading (**B**) and slurry (**C**), hydroxy-propyl-β-cyclodextrin (**D**), and EOO-hydroxy-propyl-β-cyclodextrin inclusion complexes prepared by kneading (**E**) and slurry (**F**).

Diffractograms of the same compounds and inclusion complexes are illustrated in Figure 4. The β-CD crystalline profile is revealed by the presence of intense and narrow peaks, while HP-β-CD displayed broad bands, which are characteristic of amorphous substances. The disappearance of crystalline reflections and appearance of new reflections can be taken as a further confirmation of the formation of inclusion complexes [38].

A comparison of the diffractogram of EOO-β-CD complex prepared by kneading (Figure 4B) with that of β-CD (Figure 4A) points out the appearance of a peak at 6.67°, the disappearance of β-CD peaks at 8.98 and 10.60°, a shift of the peak at 12.44 to 12.88°, and a decrease in its intensity. Even though the profile of EOO-β-CD complex prepared by slurry (Figure 4C) was similar, the new peak appeared at 6.78°. 

The HP-β-CD diffractogram (Figure 4D) is characterized by two wide bands, with the latter lying at around 19° and being more evident, similarly as found by Medarević et al. [34]. EOO-HP-β-CD complexes prepared by either kneading (Figure 4E) or slurry (Figure 4F) exhibited a slight band shrinkage and lengthening compared with HP-β-CD, but maintained its characteristic amorphous structure, like that observed by SEM for complexes.

#### 2.3.4. Thermogravimetry Analysis (TG/DTG)

**Figure 5:**
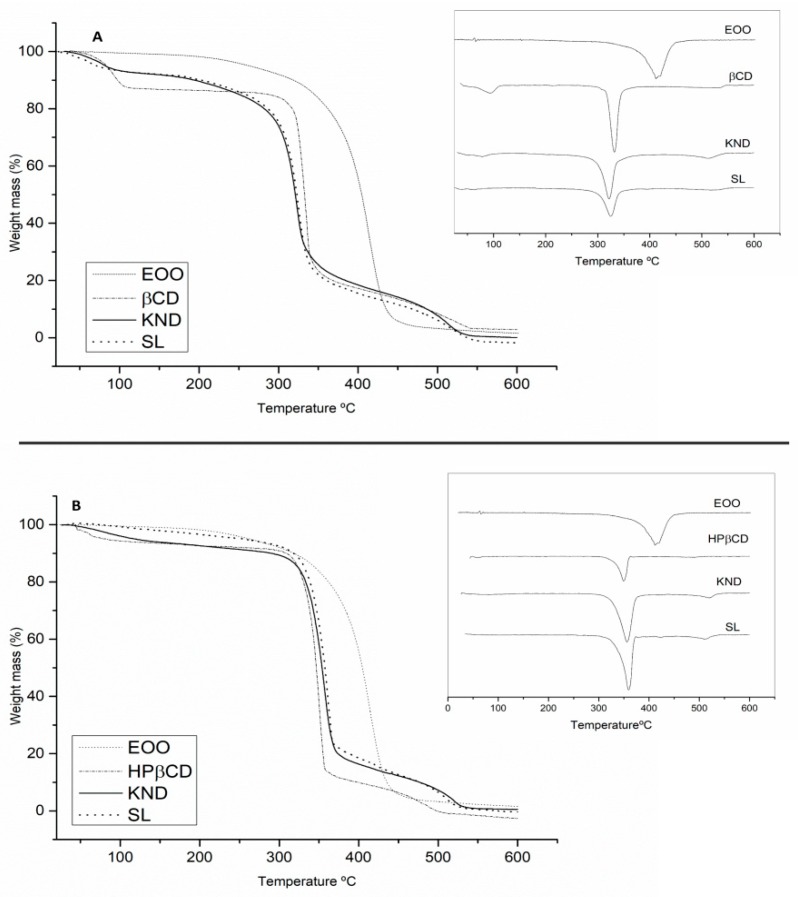
TG/DTG curves of *E. oleracea* oil (EOO), β-cyclodextrin (β-CD), and EOO-β-cyclodextrin inclusion complex prepared by kneading (KND) and slurry (SL) (**A**). TG/DTG curves of EOO, hydroxy-propyl-β-cyclodextrin (HP-β-CD) and EOO-hydroxy-propyl-β-cyclodextrin inclusion complex prepared by KND and SL (**B**).

The results of TG/DTG analysis are shown in Figure 5 and Table 2. EOO displayed a single mass loss that can be associated with evaporation and/or pyrolysis of triglycerides. The event related to mass loss occurred in the range of 200–400 °C with 95.01 % mass loss (Figure 5A,B). β-CD (Figure 5A) exhibited mass losses of 13.60 and 69.18 % in the ranges of 40–200 and 200–400 °C, respectively, which were likely associated with the release of surface water molecules followed by degradation [39]. Curves of EOO-β-CD complexes prepared by KND and SL showed at 40–200 °C mass losses of 7.19 and 7.39% (Figure 5A), respectively, suggesting water loss from cyclodextrin since EOO did not lose mass in that range. The complex prepared by the latter method was also shown to be more resistant to degradation at high temperatures (200–400 °C) (Δm = 58.97%).

The TG/DTG curve of HP-β-CD (Figure 5B) revealed 4.60% mass loss in the 40–200 °C range, associated with the release of water molecules from the surface of HP-β-CD, followed by sample degradation with a loss of 82.46% in the range 200–400 °C. Whereas the EOO-HP-β-CD complex prepared by KND (Figure 5B) showed a 5.45% mass loss, that prepared by SL no mass loss at 40–200 °C but higher mass loss at 200–400 °C (Δm = 74.00% and 64.46%, respectively), and EOO no mass loss at 40–200 °C. Thus, the formation of the EOO-β-CD and EOO-HP-β-CD complexes was evidenced for both methodologies.

#### 2.3.5. Differential Scanning Calorimetry (DSC)

**Figure 6:**
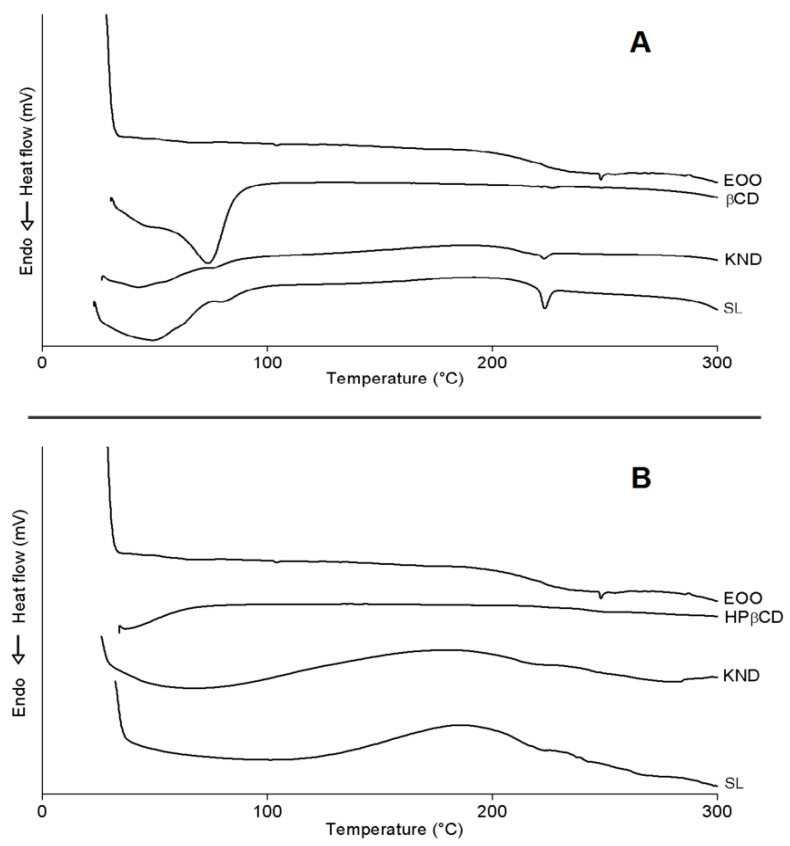
DSC curves of *E. oleracea* oil (EOO), β-cyclodextrin (β-CD) and EOO-β-cyclodextrin inclusion complexes prepared by kneading (KND) and slurry (SL) (**A**). DSC curves of EOO, hydroxy-propyl-β-cyclodextrin (HP-β-CD) and EOO-hydroxy-propyl-β-cyclodextrin inclusion complexes prepared by KND and SL (**B**).

DSC curves of all preparations are illustrated in Figure 6. One can see the occurrence of three endothermic events for EOO, the first involving a reaction enthalpy (ΔH) of 0.04 J/g with onset (Tonset) and peak (Tpeak) temperatures of 101.31 and 103.96 °C, respectively, the second ΔH = 0.14 J/g, Tonset = 241.64 °C and Tpeak = 248.96 °C, and the third one ΔH = −0.02 J/g, Tonset = 285.40 °C and Tpeak = 287.15 °C. The first two events did not show significant variations, while the third one suggests evaporation and pyrolysis of fatty acids, corroborating with oil degradation with Tonset = 239.00 °C and Endset = 444.76 °C. (Figure 5).

β-CD exhibited an endothermic peak (Figure 6A) involving ΔH = 231.02 J/g with Tonset = 30.94 °C and Tpeak = 73.71 °C, likely due to water release from its cavity, and a second event occurred at Tonset = 223.50 °C (ΔH = 0.75 J/g and Tpeak = 226.68 °C), similarly to the observations of Nascimento et al. [3]. It was not possible to identify eventual endothermic events related to β-CD mass loss at high temperatures [2,40] because a maximum temperature of 300 °C was set in this study. β-CD complexes obtained by KND and SL showed similar behavior (Figure 6A) with Tonset = 27.07 °C (ΔH = 48.46 J/g and Tpeak = 42.72 °C) and 24.07 °C (ΔH = 142.10 J/g and Tpeak = 48.86 °C), respectively, both related to dehydration, but occurring at lower temperatures when compared to β-CD. Another event characteristic of β-CD stood out between 220 and 226 °C for complexes, whose intensity was higher for that prepared by SL (ΔH = 9.8 J/g) when compared to KND (ΔH = 2.19 J/g). This difference in enthalpy may have been related to the reduction of β-CD crystalline profile, especially in the case of the latter complex; in addition, the characteristic peak of EOO at Tpeak = 248.96 °C disappeared in these curves. Both results suggest the formation of inclusion complexes.

Similarly to what observed by Pinheiro et al. [2], HP-β-CD showed an endothermic peak (Figure 6B) with Tonset = 37.25 °C (ΔH = 6.47 J/g and Tpeak = 37.05 °C) related to water release from its surface, and a second event with Tonset = 240.39 °C (ΔH = 1.98 J/g and Tpeak = 264.92 °C) related to the onset of mass loss, starting at 241.89 °C on the TG curve (Figure 5B). HP-β-CD complexes obtained by KND and SL behaved similarly (Figure 6B) with Tonset = 28.49 °C (ΔH = 60.40 J/g and Tpeak = 68.32 °C) and Tonset = 37.33 °C (ΔH = 35.79 J/g and Tpeak = 101.53 °C), respectively, both related to dehydration. Another event characteristic of HP-β-CD stood out between 234.35 and 249.41 °C, with no variation in peak intensity attributable to the preparation method (ΔH = 0.07 J/g). It was also observed in this temperature range a characteristic peak of EOO at 248.47 °C (ΔH = 0.14 J/g). 

Bearing in mind that DSC is an important analytical tool for analyzing complexation between drugs and cyclodextrins, any variation in peak temperature or intensity may be ascribed to the formation of inclusion complexes [41]. Therefore, in agreement with the other analyses, the DSC results confirm the formation of inclusion complexes by both cyclodextrins.

### 2.4. Minimum Inhibitory Concentration (MIC)

The rational use of antibiotics and the discovery of new drugs are pointed out as strategic actions to avoid bacterial resistance [42,43]. For this purpose, the Minimum Inhibitory Concentrations (MIC) of EOO, EOO-β-CD and EOO-HP-β-CD, which are indicative of their capability of preventing bacterial activity, were visually determined by the microdilution method in wells.

It can be seen in Table 3 that the highest MIC values, which are associated with the lowest bactericidal power, were obtained using EOO against *Staphylococcus aureus* (512 µg/mL), *Enterococcus faecalis* (≥1024 µg/mL) and *Escherichia coli* (512 µg/mL), while both EOO-HP-β-CD complexes obtained by KND and SL exhibited their highest efficacy against *E. coli* (MIC = 341 µg/mL) and the lowest against *Pseudomonas aeruginosa* (MIC = 512 µg/mL). However, the best performance was obtained with complexes prepared using β-CD as a carrier, especially the EOO-β-CD one obtained by SL, whose MIC values against *E. faecalis*, *P. aeruginosa*, *S. aureus*, and *E. coli* were as low as 341, 384, 384, and 256 µg/mL, respectively. Nonetheless, the EOO-β-CD complex prepared by KND was the most effective against *S. aureus*, with a MIC value of 256 µg/mL.

### 2.5. Drug Modulating Activity

There is a growing number of studies on the discovery of new antimicrobial drugs as well as the improvement of existing formulations [44,45,46,47]. Drug-modulating activity subsidizes a combination therapy that does not always improve clinical outcomes but is able to decrease the chances of bacterial resistance to multiple drugs, which is a growing problem [48].

With this aim in mind, a drug modulation assay was performed on EOO or its inclusion complexes with antibiotics commonly prescribed in clinical practice for the treatment of nosocomial and hospital diseases, in order to establish possible synergistic or antagonistic effects. Two antibiotics suggested by CLSI [49] were used against each microorganism under study, namely Ampicillin (AMP 1024 µg/mL) or Piperacillin + Tazobactam (PIP 28.5 µg/mL + TAZ 3.5 µg/mL) against *E. coli*, Ciprofloxacin (CIP 4 µg/mL) or PIP + TAZ at the same dosage against *P. aeruginosa*, and Vancomycin (VAN 16 µg/mL) or CIP 4 µg/mL against *S. aureus* and *E. faecalis.*

**Figure 7:**
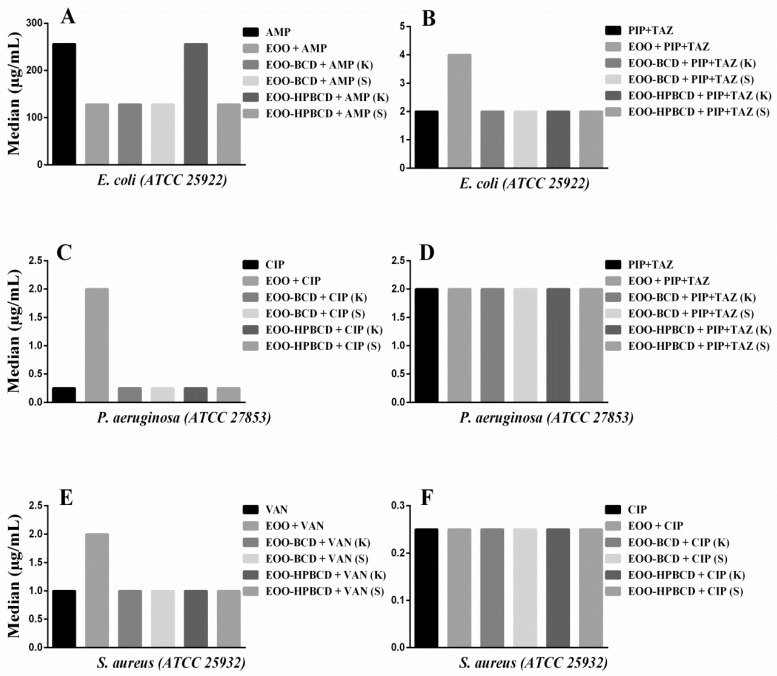
Modulatory effect of *E. oleracea* oil (EOO) and its inclusion complexes with β-cyclodextrin (EOO-βCD) or hydroxypropyl-β-cyclodextrin (EOO-HPβCD) in combination with: (**A**) Ampicillin (AMP) and (**B**) Piperacillin + Tazobactam (PIP + TAZ) against *E. coli*; (**C**) Ciprofloxacin (CIP) and (**D**) PIP + TAZ against *P. aeruginosa*; (**E**) Vancomycin (VAN) and (**F**) CIP against *S. aureus*, compared with the effect of these antibiotics alone. Inclusion complexes were prepared by either kneading (K) or slurry (S). Differences compared with the control were considered statistically significant when *p* < 0.05.

The results of modulatory activity against *E. coli* (Figure 7A) evidenced a decrease in the MIC of AMP from 256 to 128 µg/mL when this antibiotic was used in combination with EOO, EOO-β-CD prepared by either method and EOO-HP-β-CD prepared by slurry, highlighting a synergistic effect, while EOO-HP-β-CD prepared by kneading did not influence its activity. Such a synergistic effect was unexpected since the cell wall of Gram-negative bacteria is more complex than the one of Gram-positive bacteria, which has a greater number of peptidoglycan layers directly linked to penicillin-binding proteins [50,51]. Similar synergism was reported for the combination of *Croton limae* essential oil and neomycin against another strain of the same bacterial species [51]. The addition of EOO to PIP + TAZ increased the MIC from 2.0 to 4.0 µg/mL (Figure 7B), suggesting antagonistic activity, possibly related to oil solubility. Regarding the addition of inclusion complexes to PIP + TAZ, there was no significant variation in MIC.

On the other hand, an increase in MIC of CIP from 0.25 to 2.0 µg/mL was observed when used against *P. aeruginosa* (Figure 7C) in combination with EOO-HP-β-CD prepared by kneading, hence displaying an antagonistic action. This biological response suggests a modification of physicochemical properties of the antibiotic induced by such a large inclusion complex affecting its antibacterial activity [52]. On the other hand, the other preparations did not show any statistically significant difference compared with CIP alone. Moreover, since the addition of EOO and inclusion complexes to PIP + TAZ did not lead to any MIC variation compared to the administration of the antibiotic alone (Figure 7D), this suggests that the above-supposed modification was antibiotic-dependent.

Although the results against *S. aureus* (Figure 7E) showed an increase in VAN MIC from 1.00 to 2.0 µg/mL when EOO was applied in combination, there was no statistically significant effect of the inclusion complex in addition to the drug alone. The antimicrobial response of CIP against the same bacterial strain was also maintained in the presence of EOO and its inclusion complexes (Figure 7F). Since the results of modulatory activity against *E. faecalis* did not show any statistically significant VAN and CIP MIC variation induced by EOO or inclusion complexes, they were not illustrated in the figure.

Taking these results together, we can conclude that, despite the EOO ability to form inclusion complexes and improve antibacterial activity expressed as MIC, the use of cyclodextrins synergistically improved only the modulatory effect of AMP against *E. coli*, thereby strengthening the conclusions of Oliveira et al. [52]. For the other drugs, there was no significant difference in the modulatory response when used in combination with EOO and inclusion complexes, maintaining the biological response of the antibiotic.

## 3. Materials and Methods 

### 3.1. Materials

The *Euterpe oleracea* Mart (açai) oil (EOO) was obtained from Amazon Oil (Belém, PA, Brazil). This company reports that the açai fruits were collected in the Brazilian Amazon rainforest. The EOO, obtained from the fruit pulp, was extracted through a cold pressing method without the addition of solvents or preservatives. The selected fruits were washed and immersed in water at 45 °C for 60 min and processed with the aid of a stainless-steel stripper. The açai pulp was dried at a temperature of 60 °C and subjected to mechanical pressing (LDS R 135/2008, 240 kg·h^−1^) at a temperature of 40 °C. β-cyclodextrin (β-CD), hydroxypropyl-β-cyclodextrin (HP-β-CD) and resazurin were purchased from Sigma-Aldrich (St. Louis, MO, USA) and Brain Heart Infusion (BHI) broth from Kasvi (São José dos Pinhais, PR, Brazil). The ATCC bacterial strains were donated by the Microbiology Laboratory of the Federal University of Campina Grande (Cajazeiras, PB, Brazil). All experiments were performed using purified water (<1.3 μS) obtained by reverse osmosis, and the reagents were of analytical grade.

### 3.2. Chemical Characterization of Euterpe oleracea Mart Oil

EOO fatty acids composition was determined after their conversion into methyl esters (FAMEs) by saponification with 0.10 M KOH in methanol and esterification with 0.12 M HCl in methanol [53]. FAMEs were detected using a gas chromatograph, model CP 3380 (Varian, Palo Alto, CA, USA), equipped with a flame ionization detector and a CP-Sil 88 capillary column (length 60 m, internal diameter 0.25 mm, thickness 0.25 mm (Agilent Technologies, Palo Alto, CA, USA). The operating conditions were: Helium as a carrier gas at a flow rate of 0.9 min^−1^, FID detector temperature of 250 °C, injector split ratio of 1:100 and temperature of 250 °C, injection volume of 1 μL. The temperature was initially kept at 80 °C for 4 min and subsequently increased to 205 °C at 4 °C/min. Peaks of individual FAMEs were identified by comparison of retention times with those of standard solutions. The results were expressed as relative percentages of total fatty acids.

### 3.3. Preparation of Inclusion Complexes

Inclusion complexes of EOO with either β-cyclodextrin (EOO-β-CD) or hydroxypropyl-β-cyclodextrin (EOO-HP-β-CD) were prepared by two methods, namely kneading (KND) and slurry (SL), using the molar ratio of 1:1 (EOO:CD). The molecular weight of EOO was considered the same as oleic acid (282.47 g·mol^−1^) as it was its major component, while the molecular weights of β-CD and HP-β-CD were assumed to be 1134.98 and 1396 g·mol^−1^, respectively, as proposed by Pinheiro et al. [2].

#### 3.3.1. Kneading

The KND method was developed as described by Pinheiro et al. [2] with modifications in the water volume and temperature utilized. Briefly, complexes were prepared by homogenizing EOO with either cyclodextrin and adding 2.0 mL of water to form a paste. Samples were dried at 70 °C, crushed, and stored in hermetically-sealed glass ampoules in a desiccator.

#### 3.3.2. Slurry

The SL method was developed as described by Quintans-Júnior et al. [54]. Complexes were prepared by homogenizing EOO with either cyclodextrin and then gradually adding water in a 3:4 (*v*/*w*) ratio. Samples were then agitated at 400 rpm with a magnetic stirrer for 36 h. Mixtures were kept in a hot air circulating oven at 70 °C for 24 h and then conditioned in a desiccator until complete drying.

### 3.4. Determination of the Interaction Energy

To determine the interaction energy between oleic acid, i.e., the main EOO component, and β-CD or HP-β-CD, two molecular models were tested using the UCSF-Chimera software (San Francisco, CA, USA) [32], one with oleic acid entering through the wider face of cyclodextrin and the other through its narrower face. The molecules topologies were generated by Automated Topology Builder (ATB; version 2.2) and Repository [55].

Molecular dynamics were performed with the GROMACS program (Groningen, Netherlands) [56] after energy minimization of the inclusion complexes, at pH 7.4 and using TIP3P as a solvent model. Systems were maintained under isobaric (1 bar) and isothermal (300 K) conditions. The GROMOS 53A6 force field was considered for all the simulations [57]. The mean energies were obtained for a 50 ns simulation stretch. To evaluate the interaction between binder and solvent, similar simulations of oleic acid were performed in the absence of cyclodextrin. Coulomb electrostatic interactions and Lennard-Jones hydrophobic interactions were used as parameters to predict the free energy of the systems according to the Linear Interaction Energy (LIE) methodology [58].

### 3.5. Physicochemical Characterization of Inclusion Complexes

Inclusion complexes were characterized by Fourier transform infrared (FTIR) spectroscopy using a spectrometer, model IR Prestige-21 (Shimadzu, Kyoto, Japan), according to the Attenuated Total Reflectance (ATR) modality. Samples were read in the wavenumber region from 4000–700 cm^−1^ with 20 scans and 4 cm^−1^ resolution.

β-CD, HP-β-CD, EOO-β-CD and EOO-HP-β-CD were then analyzed by both Scanning Electron Microscopy (SEM) and Powder X-Ray Diffraction (PXRD). After mounting samples onto aluminum foil supports (stubs), they were covered with carbon tape, coated with a thin layer of gold and examined with a HITACHI^®^ Scanning Electron Microscope model TM-3000 Tabletop (Minato-ku, Tokio, Japan) with an accelerated voltage of 20 kV and 100× magnification. Crystalline characterization was performed by PXRD on powdered samples using a D2 Phaser diffractometer (Bruker, Billerica, MA, USA) equipped with a Lynxeye detector and operating with CuKα1 radiation nickel filter (λ = 1.54 Å), 0.02° pitch, with measurement in the angular range of 3–45°, and 30 kV voltage and 10 mA current intensity.

Thermogravimetric (TG) analysis was performed on a TGA-50/50H analyzer (Shimadzu, Kyoto, Japan) in the temperature range from 25–600 °C, using platinum crucibles containing approximately 3 mg of samples, under a dynamic nitrogen atmosphere (50 mL·min^−1^) and heating rate of 10 °C·min^−1^. To obtain the TG/DTG profiles, the equipment was calibrated with calcium oxalate monohydrate, according to Santos Costa et al. [59].

For the Differential Scanning Calorimetry (DSC) analysis, a sample of approximately 5–10 mg was put into a hermetically-closed aluminum crucible and analyzed with a calorimeter, model DSC-60 (Shimadzu), under a nitrogen atmosphere at a flow rate of 50·mL^−1^ and a heating ratio of 10 °C·min^−1^ in the temperature range 25–300 °C. Mass losses and enthalpy changes were estimated using the TA 60w program (Shimadzu) as described by Aguiar et al. [60].

### 3.6. Determination of Bactericidal Activity of Inclusion Complexes

The broth microdilution method was used to quantify the bactericidal activity of inclusion complexes expressed as Minimum Inhibitory Concentration (MIC). *Enterococcus faecalis* (ATCC 29212) and *Staphylococcus aureus* (ATCC 25932) were selected as Gram-positive standard bacterial strains, whereas *Pseudomonas aeruginosa* (ATCC 27853) and *Escherichia coli* (ATCC 25922) as Gram-negative ones.

Strains were replicated in Brain Heart Infusion (BHI), and after 24 h the tests were performed. Initially, each strain was diluted in saline solution at turbidity equivalent to 0.5 on the McFarlane scale. The inoculum was added in a test tube containing 10% BHI in a 1:9 (v/v) ratio, thereby obtaining the bacterial suspension. Microdilution was then performed in 96-well plates, in which all wells received 100 μL of bacterial suspension. Samples (100 μL) of each substance (EOO, EOO-β-CD and EOO-HP-β-CD) obtained either by KND or SL were applied to the first well of each plate column, as well as control compounds, namely penicillin, ampicillin, piperacillin-tazobactam and dimethylsulfoxide (DMSO), and a serial microdilution was carried out to the penultimate well. 

The concentration of substances was 1024 μg·mL^−1^ in the first well and decreased to 8 μg·mL^−1^ in the penultimate one, while the last well received the bacterial suspension. After incubation of plates for 24 h at 37 °C, 20 μL of 0.01 (*w*/*w*) resazurin were added in each well, and turbidity was visually assessed after 1 h at room temperature. Tests were performed in triplicate and repeated three times.

### 3.7. Drug Modulation Test

The Drug Modulation Test was performed according to Oliveira et al. [54] in order to evaluate the activity of the substances under study as modulators of antimicrobial drugs commonly applied to laboratory clinical practice [49]. For this experiment, aliquots of each sample were added to each previously prepared bacterial suspension in order to achieve sub-inhibitory concentration (MIC/8). For the determination of the required sample volume, the initial concentration of 1024 μg·mL^−1^ was considered for a final volume of 100 μL per well. After inoculation of each well with 100 μL of bacterial suspension, a serial microdilution was performed with 100 μL of the solution of standard antibiotic for each strain up to the penultimate well. The final 100 μL were discarded from this microdilution. The initial concentration of antibiotics was adopted according to the microbial susceptibility and sensitivity provided in the CLSI manual [50]. The last well received only the bacterial suspension, and the following procedure was the same as for MIC determination.

### 3.8. Statistical Analysis

The antimicrobial study was expressed as means and standard deviation, and the median was considered for modulatory activity. The results were analyzed by two-dimensional analysis of variance (ANOVA) using Graph Pad Prism 6.0 (San Diego, CA, USA). Values of *p* < 0.05 were considered statistically significant. 

## 4. Conclusions

This is the first study that evaluates the antibacterial and modular activity of *Euterpe oleracea* oil (EOO) and cyclodextrin inclusion complexes. The results of molecular modeling, FT-IR, SEM, PXRD, TG and DSC analyses revealed that EOO was able to successfully form inclusion complexes, especially with β-cyclodextrin (β-CD), confirming the expectations arisen by the estimated interaction energy between oleic acid and β-CD (Δ*G* = −41.28 ± 0.57 kJ/mol). Accordingly, FT-IR showed displacements in wavenumbers and a variation in peak intensity characteristic of cyclodextrin and EOO. The morphological and crystalline characterizations showed variations in the form, appearance, and amorphous profile of the complexes compared to cyclodextrins alone. As for thermal analysis, temperature variations in mass losses and enthalpy variations between EOO, cyclodextrins and inclusion complexes confirmed the formation of inclusion complexes. Minimum Inhibitory Concentration (MIC) results showed that the inclusion complexes presented antibacterial efficiency in Gram-positive and negative strains, being considerably better compared to pure EOO. The MIC values of the complexes obtained with β-CD were lower than those of EOO and complexes with hydroxypropyl-β-cyclodextrin (HP-β-CD). Notably, β-CD inclusion complexes showed better responses regarding complexation and biological activity than those prepared using HP-β-CD as a carrier. EOO and most of its complexes showed a synergistic effect on ampicillin against *E. coli*. In addition, inclusion complexes provided physicochemical stability to the compounds and showed no loss in EOO biological efficiency, hence supporting its therapeutic applications. The antimicrobial response exhibited by the complexes was of great significance because it subsidizes studies for the development of new drugs and pharmaceutical forms.

## Figures and Tables

**Table 1:** Composition of *Euterpe oleracea* Mart oil fatty acids quantified using gas chromatography with a flame ionization detector.

Fatty Acid	Content in the Oil (%)
Oleic acid 18:1	47.58
Linoleic acid 18:2	13.58
Palmitic acid 16:0	24.06
Palmitoleic acid 16:1	6.94
Myristic acid 14:0	1.75
Lauric acid 12:0	4.77

**Table 2:** Results of thermogravimetry analysis in terms of mass loss (%) in the temperature ranges 40–200, 200–400 and 400–600 °C.

Sample	∆m_1_ (%)	∆m_2_ (%)	∆m_3_ (%)
	40–200 °C	200–400 °C	400–600 °C
EOO	-	95.01	37.67
β-CD	13.60	69.18	10.88
EOO-β-CD (KND)	7.19	70.23	14.26
EOO-β-CD (SL)	7.39	58.97	11.37
HP-β-CD	4.60	82.46	9.09
EOO-HP-β-CD (KND)	5.45	74.01	10.01
EOO-HP-β-CD (SL)	-	64.46	6.15

Abbreviations: EOO, *Euterpe oleracea* oil; EOO-β-CD, EOO and β-cyclodextrin inclusion complex; EOO-HP-β-CD, EOO and hydroxy-propyl-β-cyclodextrin inclusion complex; KND, preparation by kneading; SL, preparation by slurry.

**Table 3:** Mean values of the Minimum Inhibitory Concentration (µg/mL).

Sample	Gram-Positive Bacteria		Gram-Negative Bacteria	
	*S. aureus*	*E. faecalis*	*P. aeruginosa*	*E. coli*
EOO	512	≥ 1024	427	512
EOO-β-CD (KND)	256	480	384	341
EOO-β-CD (SL)	384	341	384	256
EOO-HP-β-CD (KND)	469	512	512	341
EOO-HP-β-CD (SL)	384	402	512	341
Ampicillin	-	32	-	48
Penicillin	0.13	-	-	-
Piperacillin + Tazobactam	-	-	2	-

Abbreviations: EOO, *Euterpe oleracea* oil; EOO-β-CD, EOO and β-cyclodextrin inclusion complex; EOO-HP-β-CD, EOO and hydroxy-propyl-β-cyclodextrin inclusion complex; KND, preparation by kneading; SL, preparation by slurry. Tests were performed in triplicate and in three repetitions. Values of *p* < 0.05 were considered statistically significant.

## Abbreviations

AMP	Ampicillin
MIC	Minimum Inhibitory Concentration
CIP	Ciprofloxacin
DSC	Differential scanning calorimetry
EOO	*Euterpe oleracea* oil
EOO-HP-β-CD	Oil and hydroxy-propyl-betacyclodextrin inclusion complex
EOO-β-CD	Oil and betacyclodextrin inclusion complex
FTIR	Fourier-transform infrared
KND	Kneaded
PEN	Penicillin
PIP + TAZ	Piperacillin + tazobactam
PXRD	Powder X-ray diffraction
SEM	Scanning electron microscopy
SL	Slurry
VAN	Vancomicine
MM	Molecular Modeling
